# Evaluation of nine statistics to identify QTLs in bulk segregant analysis using next generation sequencing approaches

**DOI:** 10.1186/s12864-022-08718-y

**Published:** 2022-07-06

**Authors:** Carla de la Fuente Cantó, Yves Vigouroux

**Affiliations:** DIADE, Institut de Recherche Pour Le Développement, Université de Montpellier, Montpellier, France

**Keywords:** BSA, NGS, Statistics, Confidence interval, QTL, BSA-Seq, Simulation

## Abstract

**Background:**

Bulk segregant analysis (BSA) combined with next generation sequencing is a powerful tool to identify quantitative trait loci (QTL). The impact of the size of the study population and the percentage of extreme genotypes analysed have already been assessed. But a good comparison of statistical approaches designed to identify QTL regions using next generation sequencing (NGS) technologies for BSA is still lacking.

**Results:**

We developed an R code to simulate QTLs in bulks of F2 contrasted lines. We simulated a range of recombination rates based on estimations using different crop species. The simulations were used to benchmark the ability of statistical methods identify the exact location of true QTLs. A single QTL led to a shift in allele frequency across a large fraction of the chromosome for plant species with low recombination rate. The smoothed version of all statistics performed best notably the smoothed Euclidean distance-based statistics was always found to be more accurate in identifying the location of QTLs. We propose a simulation approach to build confidence interval statistics for the detection of QTLs.

**Conclusion:**

We highlight the statistical methods best suited for BSA studies using NGS technologies in crops even when recombination rate is low. We also provide simulation codes to build confidence intervals and to assess the impact of recombination for application to other studies. This computational study will help select NGS-based BSA statistics that are useful to the broad scientific community.

**Supplementary Information:**

The online version contains supplementary material available at 10.1186/s12864-022-08718-y.

## Background

The outstanding progress in high-throughput genotyping technologies over the last decade has prompted new approaches for more efficient dissection of the genetic architecture of complex traits [1]. The increased mapping resolution reached thanks to deep sequencing technologies has enhanced the estimation of allele frequencies within a population and increased the power of detection of genetic variants associated with the phenotypic variation of a trait [2, 3]. Cost-efficient NGS technologies such as genotyping by sequencing (GBS) [4] significantly facilitates the identification of interesting SNPs for marker-assisted breeding programmes. For instance, marker-trait associations for agronomic traits [5] and resistance to biotic [6, 7] and abiotic [8] stresses have been identified in GBS-GWAS studies of major crops, including wheat and rice.

The same technology applied to QTL mapping studies in bi-parental populations (RILs, double haploids, etc.) has helped validate QTLs detected in association studies [9, 10], but the detection capacity of QTL mapping studies still mainly depends on the genetic architecture of the quantitative traits, the mapping resolution, and the size of the population used [11]. A simultaneous increase in population size and marker density improves QTL detection power. Larger numbers of QTLs with smaller average effects can be identified more precisely, partly due to the dissection of closely linked QTLs. However, the gains in power of QTL detection achieved by increasing the size of biparental or multiparental populations (i.e., beyond 500 segregant lines) rarely compensates for the phenotyping and genotyping effort required [12, 13]. In recent years, bulk segregant analyses (BSA) build on NGS technologies have proved to be a highly efficient strategy for QTL mapping in linkage mapping studies [14–17], and for use in GWAS analysis with diversity panels [18, 19]. The NGS-based BSA method establishes contrasted bulks of lines from a population segregating a particular trait and explores the differences in the segregation of alleles using sequencing from the bulks. Bulking pools of contrasted lines for a particular trait greatly reduces the genotyping efforts required in the segregating population (F2s, RILs, etc.). Moreover, using a BSA based approach means larger populations can be considered, which, in some studies on yeast [20] and *Arabidopsis* [21], include up to several thousand individuals. In crops, the size of the population used generally comprises a few hundred individuals [22, 23], but we found one example of very large population in which more than 10,000 F3 rice lines were screened for cold tolerance. In that study, extreme bulks of around 400 lines were used for BSA [14]. BSA can therefore select the extreme phenotypes more likely to harbour causative polymorphism more reliably. Defining the extreme phenotypes of an easily measurable trait does not require precise characterisation of individual traits [14] thus greatly reducing the phenotyping effort required.

One major challenge of NGS-based BSA studies is screening for deviations in allele frequency that are linked to QTL regions in large datasets obtained using deep sequencing technologies. Even though using high dimensional genomic data markedly increases the mapping resolution for QTL detection, it also introduces sequencing noise linked to factors such as marked variation in sequencing read coverage or the unevenness in SNP density [24]. Consequently, a significant proportion of the new statistical methods used for NGS-based BSA studies have focused on identifying smoothing methods to reduce the effects of noise and to avoid spurious QTL associations. The statistical method based on differences in allele frequency proposed by Takagi et al. [22] has become one of the most widely used approaches in the field [16, 25, 26]. Other popular methods based on G-test [27] or Euclidian distance statistics [28] to measure the allelic divergences between the bulks have also been widely applied [15, 29, 30]. The implementation of some of these methods in R packages such as QTLseqr [31] has facilitated their application. While recent studies mostly use smoothed statistics to minimise the signal from sequencing noise following the calculation of differences in allele frequencies between the bulks, the statistics remain largely dependent on the properties of the population, including population size, recombination rate or QTL effects. These effects have been less frequently considered when optimising the choice of NGS-based BSA statistics.

The objective of this work was to perform a numerical study to 1) analyse which statistical method best identifies a hypothetical QTL, 2) analyse which statistical method identifies the QTL position most accurately, 3) build confidence intervals around the QTL location. We tested nine NGS-based BSA statistics and evaluated their effectiveness for the detection of QTLs. We used simulated data to assess the impact of variations in the recombination rate. We propose a simple tool to help choose the most appropriate approach to run statistics in NGS-based BSA studies based on the characteristics of the QTL population and of the species under study.

## Results

### BSA simulation settings for three models of recombination

**Fig. 1:**
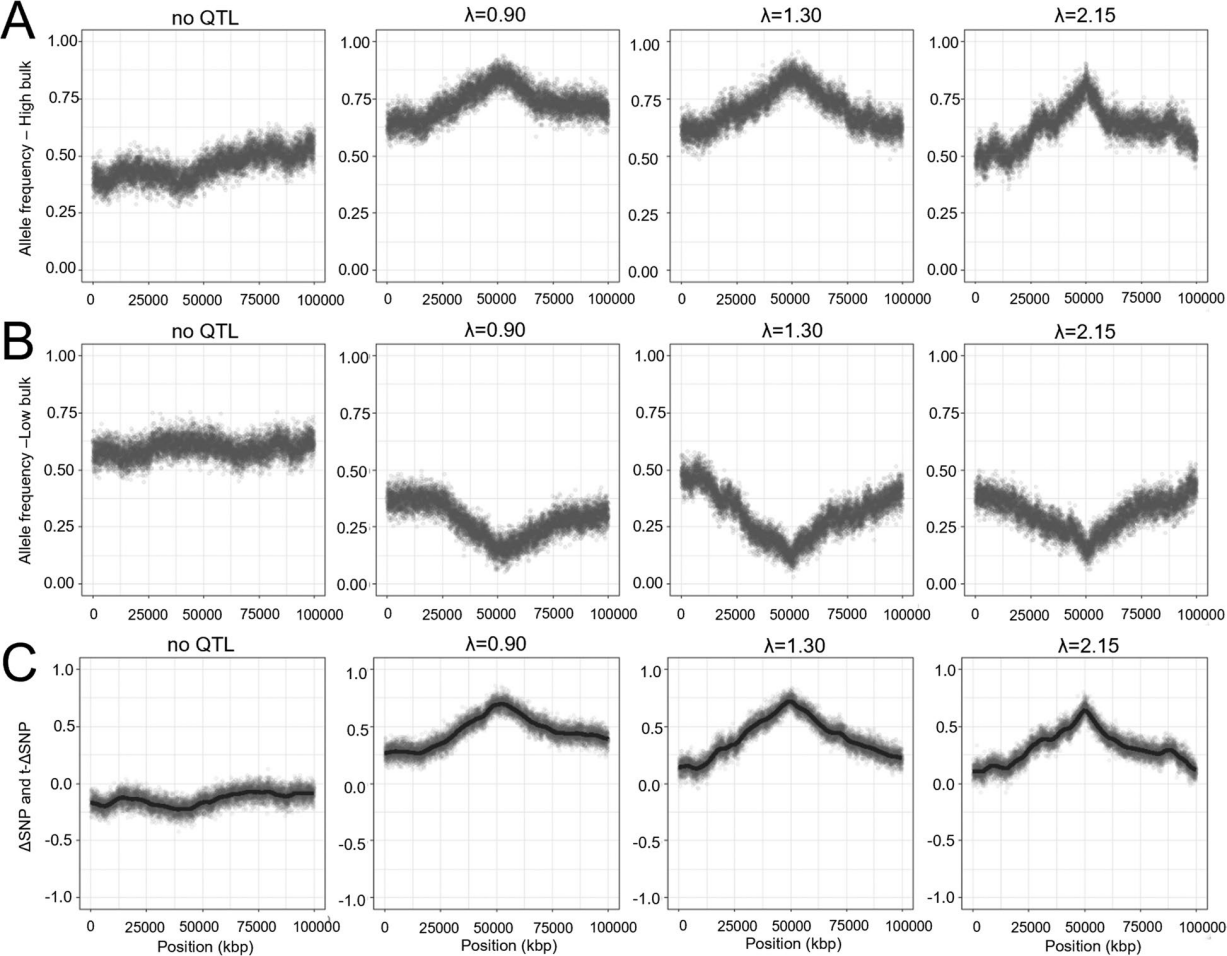
Simulation of a bulk segregant analysis with different recombination rates. The frequency of the alternate allele for each marker position is shown in two contrasted pools of segregant lines displaying high phenotype (**A**) and low phenotype (**B**). The difference in allele frequency among the pools and the smoothed statistics for the window corresponding to 3 Mbp (line) led to the detection of a QTL simulated in the middle of the 100 Mbp model chromosome (**C**) in different species with different recombination ratios (λ): pearl millet (λ = 0.90), rice (λ = 1.30) and foxtail millet (λ = 2.15). The results correspond to simulations using binomial distribution in the simulation of sequencing noise and QTL effect equivalent to 20% of the phenotypic variance (k = 1). The first graph in A, B and C shows the results in absence of a QTL effect

**Fig. 2:**
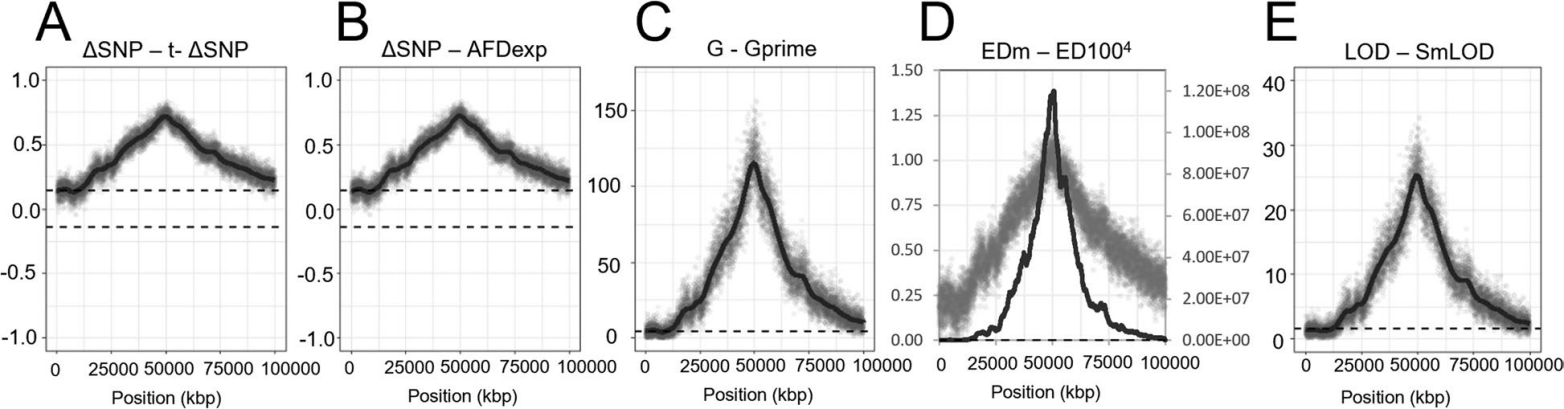
QTL detection using different statistics. The difference in allele frequency among contrasted bulks at the marker position (dots) and the corresponding smoothed statistics (line) is represented according to QTL-seq method based on ΔSNP [22] (**A**), Block Regression Mapping [32] (**B**), G statistics [27] (**C**), Euclidean distance-based statistics [28, 30] (**D**) and QTG-Seq method based in LOD statistics [30] (**E**). The dotted lines show the 95% confidence interval defined for each method. The analysis presented here was performed on rice (λ = 1.30)

We first simulated a single QTL in the middle of a chromosome and used nine statistics that are commonly used in NGS-based BSA studies to detect it (Figs. 1 and 2 and Additional file 1: Fig. S1). In the absence of loci affecting the trait, the genotype frequencies showed similar variations between the two contrasted bulks, with values around 0.5 and a difference close to zero. In contrast, the presence of a QTL linked to the phenotype led to a bias in allele frequency in the bulks. Alternate alleles were overrepresented around the QTL region in the bulk with high phenotypic values compared to in the bulk with low phenotypic values. The difference in allele frequency of the alternate allele between the bulks (∆SNP) and a smoothed version of the difference in allele frequency (t-∆SNP) revealed the presence of a QTL peak in the middle of the chromosome where the alternate alleles affect the quantitative trait positively (Fig. 1).

All nine statistics located the QTL region (Additional file 1: Fig. S1). Bigger differences in allele frequency were observed closer to the causative locus (centred in the chromosome at 50 Mbp). The plots obtained using statistics based on the difference in allele frequency (Fig. 2 A and B) produced similar results. In this case, the statistic varied between -1 and 1, indicating alleles with a positive or negative effect on the quantitative trait. BSA based on the G statistic and LOD score produced similar QTL detection plots (Fig. 2 C and E). These two statistical tests only indicate whether the alleles have an effect on the quantitative trait. In these four analyses, windows equivalent to 3 Mbp were found to efficiently smooth the noise that resulted from sequencing. The value of the smoothed statistic is the result of a function (Nadaraya-Watson kernel regression or Loess regression) that calculates a weighted average of the statistics across the SNPs within a sliding window with a given physical distance. Finally, the statistic based on Euclidean distance successfully detected the QTL. In this case, the smoothed statistic computed based on the fourth power of hundred consecutive markers accentuated the QTL peak signal better than the other methods. In parallel simulations, we used high coverage sequencing data from African rice [33] to add sequencing noise to the simulation of the reference and alternate allele depth based on a real dataset and compared the results with our theoretical approach based on a binomial distribution. We observed a similar trend in the results obtained with a slightly higher variance in allele frequency and therefore in the value of the statistic at each marker position (Additional file 1: Fig. S2). Despite the increased noise, the smoothed statistics showed a very similar pattern across simulations. Finally, we also tested the detection of QTLs with a minor effect on the phenotype equivalent to roughly 6% of the phenotypic variance. We found that all statistics detected the QTL peak but with major differences among them. The Euclidean distance smoothed statistics still led to a more accurate detection of the QTL peak in the three recombination models (Additional file 1: Figs. S3 and S4).

High-density marker coverage reveals the effect of the recombination rate on the segregation of alleles in the bulks. In low recombinant species such as pearl millet (λ = 0.90), the QTL effect seems to spread across a large fraction of the chromosome. By contrast, rice (λ = 1.30) and the highly recombinant model foxtail millet (λ = 2.15) showed strongly marked peaks at the position of the QTL. The same trend was observed in the output of the five statistical methods (Additional file 1).

### QTL identification and the effect of the recombination rate

**Fig. 3:**
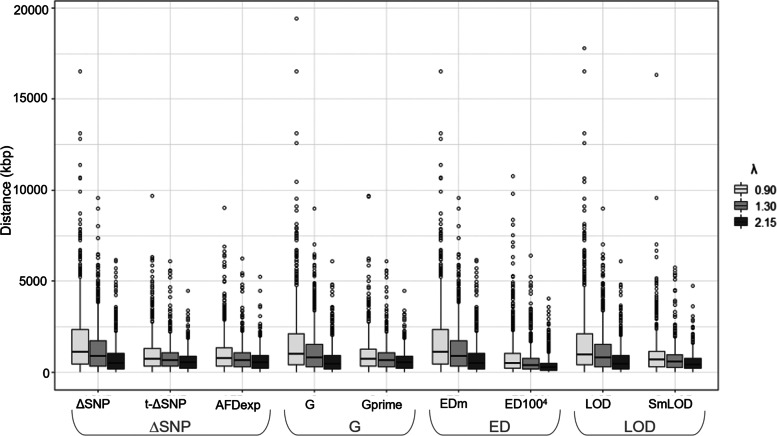
Comparative analysis of the inferred position of simulated QTLs. To assess the accuracy of QTL detection using nine statistics based on calculation of ∆SNP, G, ED and LOD at the marker level in three case studies of the recombination rate (λ), we plotted the distance to the simulated QTL in kb. Our calculations were based on differences in allele frequency (∆SNP), G-statistics (G), Euclidian distance (ED) and log likelihood (LOD). Methods use either data at the SNP level (∆SNP, G, EDm, LOD) or a smooth value across several SNPs (t-∆SNP, AFDexp, Gprime, ED100^4^, SmLOD). The boxplots represent the range of detection of QTLs in one thousand simulations using each method. Distance corresponds to the absolute genetic distance between the simulated QTL position and the QTL position retrieved with each method. Binomial distribution was taken into consideration in the simulation of sequencing noise

The accuracy of QTL detection was assessed as the distance between the initial QTL position and the position identified with each statistic. The mean distance was estimated from one thousand simulations for each statistic and recombination model (Fig. 3) as well as for simulations showing a slight increase in sequencing noise as a consequence of using real rice sequencing data (Additional file 2: Fig. S1). All the methods detected a significant QTL at the desired location, but the accuracy of the location varied across the nine statistics tested and depending on the recombination rate, especially in simulations that used the binomial function in the definition of sequencing noise (Additional file 2: Table S1). In cases with a slight increase in sequencing noise (i.e., simulations based on real data) the differences among the statistics in the accurate detection of QTL was remarkably independent of the recombination model (Additional file 2: Table S3). Still, in all the simulations, the higher the recombination, the more accurate the detection of the QTL. In pearl millet (λ = 0.90), the QTL was located between 1,594 kb (G statistics) and 1,729 kb (ΔSNP and EDm statistics) average distance from the original QTL position considering SNP based statistics. This average distance was reduced with smoothed statistics and ranged between 863 kb (ED100^4^) and 1,158 kb (AFDexp). To give an approximate number of genes, a 863 kb distance to the QTL in pearl millet (ED100^4^) is equivalent to a “distance” of 20 genes from the true QTL. To perform this approximate calculation, we assumed the 38,579 genes in the 1.7 G pearl millet genome are equally distributed on its seven chromosomes. These values showed that smoothing statistics increased the accuracy of QTL detection, particularly Euclidean distance, when the recombination rate is low (Additional file 2: Table S2). Similar results were obtained when real data was used to simulate sequencing noise. In this case, the average distance to the original QTL position ranged from 1,993 kb (ΔSNP and EDm statistics) to 3,510 kb (LOD statistic) when SNP based statistics were used in the plant species with low recombination rate (pearl millet, λ = 0.90). Smoothed statistics reduced this average distance to values ranging from 913 kb (ED100^4^) to 1,047 kb (AFDexp) (Additional file 2: Table S4). By contrast, the QTL positions detected in the foxtail millet model study, which have a higher recombination rate (λ = 2.15), were more precisely located, independently of the statistic used (Additional file 2: Table S1). The average distance ranged between 629 kb (G statistics) and 777 kb (ΔSNP and EDm) with SNP based statistics; between 402 kb (ED100^4^) and 822 kb (AFDexp) with smoothed statistics (Additional file 2: Table S2). These values ranged between 901 kb (ΔSNP and EDm) and 1,805 kb (G) with SNP based statistics using real data to simulate sequencing noise, between 428 kb (ED100^4^) and 653 kb (Gprime) when using smoothed statistics (Additional file 2: Table S4). Interestingly, the SNP based statistics G and LOD varied more in simulations based on real data (i.e., higher sequencing noise). In this case, the smoothed statistics, Gprime and SmLOD, greatly improved the accuracy of QTL detection in all the recombination models (Additional file 2: Fig. S1).

**Fig. 4:**
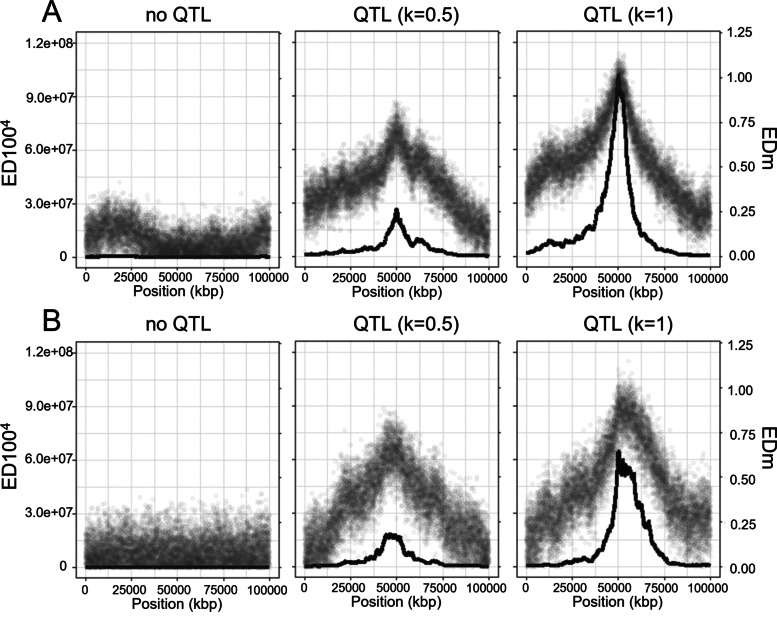
NGS-based BSA case study in the absence of a QTL effect and with a QTL effect equivalent to 5.9% (k = 0.5) and 20% (k = 1) of phenotypic variance. Results using binomial distribution (**A**) and real data from rice (**B**) to simulate sequencing noise. In each graph, the grey dots correspond to the statistics value at marker level (i.e*.*, EDm); the black line shows the smoothed value of the statistic (i.e*.*, ED100^4^)

Remarkable differences were also found within each group of statistics. The statistical tests at SNP level (∆SNP, G, EDm and LOD) gave a less accurate estimation of the QTL position than the smoothed version with the same statistic (t-∆SNP and AFDexp, Gprime, ED100^4^ and SmLOD). The biggest differences were found for the Euclidean distance-based statistics in all recombination models. The smoothed version of the statistics, ED100^4^, remarkably improved the QTL detection in the three recombination models with the distance to the causative locus reduced by half compared with SNP based statistics, EDm (from 1,729 kb to 864 kb for λ = 0.90; from 1,233 kb to 608 kb when λ = 1.30 and from 777 to 403 kb when λ = 2.15). The smoothed version of ΔSNP (t-ΔSNP) also improved the accuracy of the QTL detection by reducing the average distance to the causative SNP by 44% (λ = 0.90), 35% (λ = 1.30) and 19% (λ = 2.15). Finally, smoothed G statistics improved QTL detection and yielded distances 40% and 30% closer to the causative locus when λ = 0.90 and λ = 1.30 respectively and 7% closer when λ = 2.15 (Additional file 2: Table S2). The increased efficiency of smoothed statistics coincided with a reduced confidence interval width for QTL detection, which was up to 25% smaller with the Gprime and SmLOD statistics compared to G and LOD, respectively. The confidence intervals of the statistics were found to have the same range of variation across the different recombination models using 10,000 simulations (Additional file 3). When we compared QTLs with low effect (roughly 6% compared to 20%), again we found that ED100^4^ distinguished a pattern with no QTL and with a QTL effect^.^ (Fig. 4). This pattern was also rather similar whether we considered noise based on binomial law or based on real rice sequencing data. In conclusion, even in the case of a low QTL effect, ED100^4^ performed remarkably better than the other statistics.

## Discussion

### NGS-based BSA statistical methods for large complex datasets

Combining NGS technologies with Bulk Segregant Analysis (BSA) increases the power and efficiency of linkage mapping remarkably, thus providing a great opportunity to accelerate gene identification and QTL mapping in a cost-efficient way. Since BSA was first developed in the early 1990s [34, 35], the marker densities obtained in high-throughput genotyping technologies have steadily increased, accompanied by significant increases in population size and phenotyping throughput. Altogether, the resolution of QTL mapping in NGS-based BSA studies has increased remarkably and enhanced the power of QTL detection compared with conventional linkage mapping approaches [3, 14–16, 36, 37].

Statistical approaches for NGS-based BSA studies have also been adapted to be able to deal with the complexity of large marker datasets and the inherent sequencing noise that may mask true genetic diversity, thereby distorting the results of genetic studies [38]. Smoothed statistics in particular have proved to be effective in improving the accuracy of QTL detection whilst taking linkage disequilibrium between SNP markers into account [27, 31]. However, one major limitation of smoothed statistics tools is their dependence on parameters, for example, on window width, whose value is not easy to determine [27]. A great number of approaches and tools are available, and it is not always clear which is the most suitable for a given study.

Here, we performed a computational study to compare the performance of nine statistics for NGS-based BSA studies used for the genetic dissection of quantitative traits. Four of the statistics we tested measure the difference in allele segregation between the bulks at single marker level (delta-SNP, G, EDm and LOD). The other five methods tested used a smoothing analysis based on sliding windows and a weighted average for a given physical distance [22, 27, 30, 32] or fixed windows for a defined number of consecutive markers [30].

Our results confirm that smoothed statistics dealt best with the unevenness of sequencing data in the estimation of differences in allele frequency between bulks. The five smoothed statistics increased the accuracy of QTL detection compared with the corresponding marker-based statistic. In our model study, we assumed that marker density was homogeneous along the chromosome. Consequently, the two smoothing approaches predicted a similar QTL position. However, in real datasets, fixed window width might be more sensitive to skewing in SNP density along the chromosome, particularly in regions with low marker density. In this case, nonparametric fitting methods for a given genetic distance may be more appropriate to smooth datasets with big differences in marker density along the genome [28].

### Optimisation of NGS-based BSA analysis using computational methods

The structure and size of the population are major factors to consider in the optimisation of statistical approaches for NGS-based BSA studies [39]. Here, we used a standard approach and simulated a population of 500 segregant diploid F2 lines (50 lines per bulk) to test the accuracy of different statistics and to emphasise differences when applied to a relatively small population for QTL fine-mapping [40]. NGS-based BSA studies are frequently performed using early generations from biparental crosses (F2s, F3s) for reasons of cost efficiency [17]. These studies generally combine a BSA analysis with other QTL linkage mapping approaches [15, 25, 30, 41] or with a more extensive GWAS analysis [42, 43]. Using RILs from the F6 or F7 generation improves the power of QTL detection of NGS-based BSA [22], but carrying out the additional crosses is labour intensive and may only offset the cost of NGS-based BSA when applied to inbred species with large complex genomes like wheat [23, 44].

In addition, larger populations increase the probability of having enough recombination to enclose QTLs in smaller genomic regions. NGS-based BSA studies in very large populations (~ 100,000 individuals) of yeast [20] and *Arabidopsis *[21] showed the marked impact of increasing population size on enhancing the power of detection of small and large effect QTLs as well as in resolving linked QTLs. However, these population sizes are not easy to manage in experiments on crop species. It has also been proposed that the analytical power could be improved by increasing the number of genotypes in the bulks, but this would also increase the probable presence of intermediate phenotypes in the bulks, which in turn would negatively affect the power of QTL detection [22]. In this case, a possible alternative could be using multiple bulked samples comprising 20 to 50 individuals [22] from the phenotype tails when using large populations of around 10,000 individuals [45].

The identification of a suitable strategy to perform NGS-based BSA requires optimisation of parameters such as bulk size, allele sequencing depth, and the type of statistics. Simulation studies have proved to be extremely powerful tools to perform such optimisation before beginning experimental work. The simulation study by Magwene et al. [27] showed that bulks considering 10–20% of individuals with extreme phenotypes from large populations (~ 1,000 individuals) maximises the power of QTL detection in NGS-based BSA studies as long as the coverage depth is sufficient (allele depth greater than the size of the bulks). Most of the NGS-based BSA studies in crop species use this value as a reference to define the bulks [17]. In addition, increasing sequencing depth rather than marker density (beyond 0.2 per cM) seems to enable greater gains in power of QTL detection in NGS-based BSA studies [46]. Thus, an efficient strategy could consist in exploiting deep sequencing of genetic libraries representing a reduced part of the genome [18, 46–48] or targeting the transcriptome [28, 44, 49, 50] of contrasted bulks. In the counterbalance between bulk size and coverage, favouring larger bulk sizes to the detriment of the depth of sequencing may lead to greater power of detection of QTLs [51]. However, other studies suggest that considering larger bulks may only be advantageous when combined with increases in sequencing depth [27, 36]. Here, the analysis was performed with a fixed number of individuals in the population (500), a fixed size for the bulks (50), and an average coverage of 100 reads to compare the different statistics. We cannot comment further on the trade-off between bulk size and depth of sequencing from the simulation presented here. However, in future work, the tools developed in this study could be used to run a broader range of simulations to test QTL detection considering these factors in combination with others such as QTL effects or the recombination rate of the population.

### Recombination rate in the mapping population should be taken into account in the design of the experiment and in the selection of statistics for NGS-based BSA studies

NGS-based BSA studies often use empirical data and preliminary simulations to optimise experiments and processing pipelines [18, 22, 27, 30, 42, 46, 52, 53]. Studies often test parameters such as smoothing window size, population size, sequencing depth, QTL effect size, or heritability. The study by Guo et al. [46] also used the recombination rate to select methods based on G statistics and to resolve genetic linkage between flanking QTLs. As a result, the authors achieved similar power of QTL detection in empirical data as that achieved in studies that used populations about ten times larger [54]. Despite the direct influence of the recombination rate on the frequency of allele segregation, this factor is rarely taken into account in the selection and implementation of statistics for NGS-based BSA studies. In fact, it is still not clear if, in practice, recombination rates can be used to increase statistical power.

In our simulations, we tested the power of different statistics for QTL detection in three case studies in which the recombination rate varied. We used an average recombination rate across the chromosome, whereas variable recombination rates along the chromosome likely combine the average results of our three simulated scenarios (low, average, high). The results suggest remarkable differences in the power and accuracy of QTL detection using different statistics, especially when the recombination rate is low. The increased uncertainty in the position of a QTL is often due to larger blocks of linked markers with fewer crossovers in the bulks established in studies using plant species with low recombination rate such as pearl millet. In comparison, species with a higher recombination rate showed that QTL detection could be more accurate than expected [11]. Interestingly, the nine statistics used in our NGS-based BSA simulation study varied significantly in their ability to detect QTLs, especially in plant model species with low recombination rate. Overall, the statistics based on Euclidean distance proved to be more efficient in detecting QTLs across all three case studies and proved to be the most suitable when the recombination rate is low. In fact, a recent study in rice confirmed this observation using empirical data. A grain size QTL located in an 11.31 Mbp region using the QTL-seq method based on ΔSNP [22] was delimited to a 3.26 Mbp region using the Euclidean distance based method [55].

The popular QTL-seq method based on ΔSNP [22] and the G statistics method [27] have been the most widely used methods in the last decade [14, 25, 29, 56]. An alternative version of the ΔSNP statistic considers its absolute value [57] or its fourth power [29] when sequencing data from a reference parent line is lacking. However, in our simulation study and in the same settings, these methods were less precise in detecting the location of QTLs with intermediate values between Euclidean distance-based statistics and the Block Regression Mapping method (BRM) [32]. A recent study suggests that the efficiency of ΔSNP and G based statistics in detecting QTLs relies to a great extent on having relatively high sequencing coverage [58]. The authors propose an alternative approach to improve these methods and to increase the sensitivity of QTL detection in the case of lower coverage. Similarly, the BRM method based on ΔSNP as a single marker based statistic [32] is designed to cope better with low coverage datasets than the QTL-seq method [22] and G statistics [27]. Yet, in our simulation study, the BRM method produced the least precise QTL location. Smoothing based on blocks of markers equivalent to the same sized window across recombinant models was less effective in the highly recombinant model. In this case, considering recombination rates to define smaller blocks as genetic units would produce better results. Finally, in our simulations, the QTG-Seq method based on LOD statistics [30] outperformed both the QTL-seq [22] and G statistics [27], and produced results close to those obtained with Euclidean distance statistics. Recent studies combining QTG-Seq and Euclidean distance statistics were found to be efficient in the QTL fine-mapping of plant height in maize [30] and mildew resistance in melon [59]. Hence, our result suggests that recombination rates should be taken into account when selecting the most appropriate statistic for QTL mapping and when optimising the parameters used for QTL mapping in NGS-based BSA studies.

### A simulation approach to calculate confidence intervals

Confidence intervals for the estimation of QTL genomic location are important parameters to define the extent of a significant region to be searched for potential candidate genes underlying trait differences. However, only a few studies have attempted to study the accuracy of confidence intervals provided by the different statistics using simulations [22, 32, 36] or using the root mean square error (RMSE) to define a standard deviation for each QTL peak [27, 46].

In our study, we used simulations to calculate an overall confidence interval for the detection of the position of QTL for each of the nine statistics tested. We computed an overall estimation of confidence intervals that is equivalent to the mean value of the 95% statistic quantile in the absence of QTL effect in 10,000 simulations. The QTL-seq based on ΔSNP statistic ^13^ uses a similar approach but introduces read depth in the simulation to compute a value of confidence interval at each SNP position [31]. We fixed the values for average depth, QTL effect, population size and bulks size and found that the confidence interval values did not differ much across the three recombination case studies. However, our simulation approach allowed us to tailor the confidence interval to each specific study by taking into consideration factors of the analysis that were beyond the scope of this study.

## Conclusion

Nine NGS-based BSA statistics were tested for the detection of QTLs using simulations in three case studies with variable recombination rates. All the smoothed statistics proved to be more accurate in locating QTLs than marker-based statistics. The recombination rate was found to have an impact on detecting the position of QTLs as less accurate results were obtained with low recombinants. Euclidean distance-based statistics were found to enhance the accuracy of QTL detection in all recombinant models, thereby enabling major gains in the low recombination case study. The present study proposes a guideline for testing the parameters best suited for the selection of an NGS-based BSA statistical method for a F2 population and for the definition of confidence intervals.

## Material and methods

### Population design and case studies

We used a standard approach for the simulation of a bulk segregant analysis for QTL mapping based on the segregation of a trait in a F2 population derived from a bi-parental cross between diploid homozygous lines. For the sake of simplicity, we focused the study in one model chromosome with 10,000 evenly distributed loci and 100 Mbp length, i.e., one marker locus every 10 kb. To begin with, we modelled the genotype considering a population size of 500 diploid individuals (i.e., 1,000 chromosomes). The occurrence of crossovers was simulated at random and independent from each other according to the Haldane map function [60]. Using this assumption, the recombination events were defined following a Poisson distribution f (n, λ) in the total number of chromosomes at a frequency equivalent to the map distance between adjacent loci, λ [61]. 0 s and 1 s were used to code the reference and alternate parental alleles, respectively.

Next, a normally distributed phenotype N (0, 1) was modelled for the group of 500 segregants. We linked the phenotype and the genotype of the lines by simulating a single QTL with a positive additive effect on the phenotype equivalent to one or half standard deviation (k = 1 or 0.5). The formula [62] relating the fraction of standard deviation k to the explained variance π is

$$\uppi =\frac{{p}\text{(1-}{p}\text{)}{\text{k}}^{2}}{p\left(1-p\right){\text{k}}^{2}+1-1/n}$$

where *p* is the allele frequency and *n* is the number of individuals; in our case, *p* = 0.5 and *n* = 500 individuals. So, for k = 1, the percentage of the phenotypic variance explained is 20% and for k = 0.5, the percentage of the variance explained is 5.9%.

Subsequently, bulks of lines exhibiting high (H) and low (L) phenotype were established by grouping the genotype from the 10% individuals (i.e., 50 individuals) on each tail of the phenotype distribution. Counts of parental alleles at each marker position were determined for each bulk and some level of sequencing noise was introduced using a binomial function B (n, P) with a number of trials equal to the maximum sequencing depth of 100 (n) and a success probability equivalent to the allele frequency calculated without noise (P). We used a second method to add sequencing noise based on a real dataset of high coverage sequencing data from 250 African rice genomes [33] (Additional file 5). The noise was added by randomly sampling sequencing depth information at 10,000 SNP random positions. We built the bulk for 100 chromosomes (the same as in our simulated bulks) using the real depth and each individual contributed randomly a 0 or 1 allele (and their number of reads) as a function of allele frequency.

Three model species differing in recombination rate were used as case studies to test the genetic conformation of diverse mapping populations in the detection of QTLs using BSA. Pearl millet (*Pennisetum glaucum* (L.) R. Br.) was used as an example of low recombinant species with an average chromosome length of 90 cM (i.e. λ = 0.90) [63] in contrast to the high recombination rate of foxtail millet (*Setaria italica* (L.) P. Beauv.) whose average chromosome is 215 cM (i.e. λ = 2.15) [64]. Rice (*Oryza sativa* L.) was included as an intermediate case study with 130 cM average chromosome length (i.e. λ = 1.30) [65].

### Statistical methods for BSA-QTL mapping

Nine statistics were used in the identification of simulated QTLs through BSA: four statistics that compute differences in allele frequency at the marker level and five statistics that add a smoothing method to estimate this difference in groups of consecutive markers (Table 1). The analysis starts by calculating the frequencies of alternate and reference alleles of the cross using the allele depth or counts of parental alleles for each bulk at each marker position. Then, four metrics are used to define the differences in allele frequencies between the contrasting bulks at the SNP level. For instance, QTL-seq [22] and Block regression mapping (BRM) [32] are based on the subtraction of the alternate allele frequency value of the low bulk from the high bulk (∆SNP). On the other hand, the method suggested by Magwene et al*.* [27] relies on the calculation of the standard G statistic (G) and the QTG-Seq method [30] computes a log likelihood statistic LOD between allele frequencies. Finally, the approach suggested by Hill et al. [28] uses the Euclidean distance between two vectors defined by the frequencies of the alternate and the reference alleles in the high and low bulks (EDm). Based on these four statistics at the SNP level, other statistics are derived by calculating a smoothed version of the statistic in a sliding window of consecutive SNPs across the genome. The tricube-deltaSNP (t-∆SNP) and Gprime are the result of computing a weighted average of the test statistic for the SNPs (∆SNP and G) within a bandwidth window equivalent to 3 Mbp. In this case, the smoothed statistics is computed using a Nadaraya-Watson or tricube smoothing kernel [66, 67] using the QTLseqr R package [31]. The QTG-Seq approach uses the same calculation to estimate the Smooth-LOD based on the LOD statistic [30]. Conversely, the BRM method [32] uses a Loess function to deal with sequencing noise in blocks of markers equivalent to 3 Mbp in size. For the Euclidean distance-based statistics, we used the fourth power of the cumulative Euclidean distance value for fixed sliding windows of one hundred consecutive SNP markers [29, 30, 59]. Selection of window size is largely dependent on the study population. The QTL signal is attenuated to counteract sequencing noise. This step could either leave some QTL peaks out or merge proximate QTLs peaks when the windows are wide or lead to a bunch of false QTL peaks when the windows are too narrow. In addition, too narrow windows may entail limitations in the computing of smoothed statistic [31]. Our selection of window bandwidth set to 3 Mbp was based on computing limitations encountered in real datasets.

**Table 1:** Statistics from the literature adapted to our simulation study

Statistic at SNP level	Smoothed statisctic	Description	Reference
ΔSNP	t-∆SNP	Allele frequency difference (ΔSNP) smoothed by tri-cube kernel function for sliding window W	[22, 31]
	AFDexp	Allele frequency difference smoothed (ΔSNP) by Loess regression function in blocks of markers of size W condidered as genetic unit	[32]
G	Gprime	G statistic value smoothed by tri-cube kernel function for sliding window W	[27, 32]
EDm	ED_100_	Fourth power of cumulative Euclidean distance at SNP level (EDm) of 100 consecutive SNPs	[28–30]
LOD	SmLOD	Maximum-likehood statistic smoothed for tri-cube kernel function for sliding window W	[30]

### QTL detection: QTL mapping across methods and definition of confidence intervals

QTL mapping was analysed using the three case studies for recombination. First, the code settings were tested by simulating a single QTL in the three model chromosomes. The QTL was placed in the middle of the model chromosome (i.e., locus 5000) and the statistical methods were used to visualise the position of the QTL and the effect of smoothed statistics (Additional files 6 and 7). Next, we assessed the efficiency of QTL detection by running one thousand independent simulations on each model chromosome (Additional files 8 and 9). In this case, a random QTL position was defined in each loop of the simulation. The absolute genetic distance between the initial QTL position and the position retrieved by each statistical method was used to compare the accuracy of QTL detection. The QTL position considered corresponds to the locus for which the maximum difference in the statistic is reached between the bulks, or the QTL peak.

In addition, statistical values from ten thousand independent simulations with no QTL effect were used to define the confidence intervals for QTL detection (Additional file 10). The 95% quantiles for these 10,000 simulations were selected as significant threshold values to estimate the confidence interval to define QTL regions of significance with each NGS-based BSA statistic. The steps followed in the simulations are summarised in Supplementary Table S4.

## Supplementary Information

**Additional file 1.** Supplementary figures showing the results of simulations of a NGS-based BSA case study using different statistics for the detection of a single QTL in a model chromosome at three recombination rates (λ=0.90, λ=1.30 and λ=2.15). **Figure S1.** Result of simulations using binomial distribution in the simulation of sequencing noise and a QTL effect equivalent to 20% of the phenotypic variance (k=1). **Figure S2.** Result of simulations using real data from rice to add sequencing noise and a QTL effect equivalent to 20% of the phenotypic variance (k=1). **Figure S3.** Result of simulations using binomial distribution in the simulation of sequencing noise and a QTL effect equivalent to 5.9% of the phenotypic variance (k=0.5). Figure S4. Result of simulations using real data from rice to add sequencing noise and a QTL effect equivalent to 5.9% of the phenotypic variance (k=0.5)

**Additional file 2. **Supplementary figure and tables showing the results of comparative analysis of BSA statistical methods and their accuracy in locating QTLs in 1,000 simulations. **Figure S1.** Comparative analysis of the inferred position of simulated QTLs using rice real data to simulate sequencing noise. **Table S1.** Pairwise comparison of different statistical approaches inferring the position of a simulated QTL. The positions used correspond to 1,000 simulations including binomial distribution to add sequencing noise (Figure 3). **Table S2.** Summary statistics of the absolute genetic distance (kb) between the simulated QTL and the QTL position retrieved for 1,000 simulations run with each statistic in each model chromosome (Figure 3). **Table S3.** Pairwise comparison of different statistical approaches inferring the position of a simulated QTL. The positions used correspond to 1,000 simulations using rice real data to add sequencing noise (Additional file 2- Figure S1). Table S4. Summary statistics of the absolute genetic distance (kb) between the simulated QTL and the QTL position retrieved for 1,000 simulations with each statistic in each model chromosome (Additional file 2- Figure S1).

**Additional file 3.** Table showing the confidence intervals of each of the statistics. The calculation is based on 10,000 simulations for each model chromosome.

**Additional file 4.** General summary of the main steps of the simulations.

**Additional file 5.** “OgOb-all-merged.DPech.recode.vcf”. vcf file containing high coverage sequencing data from 250 African rice genomes 32.

**Additional file 6.** “Simulation-Code1a.R”. R code used to compare the mapping of a single QTL using different BSA statistics in three recombination models. The QTL was placed in the middle of the chromosome and sequencing noise was simulated according to a binomial distribution.

**Additional file 7**. “Simulation-Code1b.R”. R code used to compare the mapping of a single QTL using different BSA statistics in three recombination models. The QTL was placed in the middle of the chromosome and real data from rice were used in the simulation of sequencing noise.

**Additional file 8.** “Simulation-Code2a.R”. R code used to evaluate the accuracy of QTL detection using different BSA statistics. In this case we ran 1,000 simulations. Each loop defines a random QTL position. The absolute difference between the initial position of the QTL and the position retrieved with each method was used to compare the accuracy of the different methods. This code uses binomial distribution in the simulation of sequencing noise.

**Additional file 9.** “Simulation-Code2b.R”. R code used to evaluate the accuracy of QTL detection using different BSA statistics. In this case we ran 1,000 simulations. Each loop defines a random QTL position. The absolute difference between the initial position of the QTL and the position retrieved with each method was used to compare the accuracy of the different methods. This code uses rice real data in the simulation of sequencing noise.

**Additional file 10.** “Simulation-Code3.R”. R code used to define the confidence intervals for each statistic and each recombination model. We ran 10,000 simulations with no QTL effect. The 95% quantiles were used as significant threshold values to define the confidence intervals of each statistic.

## Data Availability

The R codes are available in the additional files and in the GitHub repository https://github.com/DLFCCarla/BSA_stats/.

## Abbreviations

BSA: Bulk Segregant Analysis
NGS: Next Generation Sequencing
SNP: Single Nucleotide Polymorphism
QTL: Quantitative Trait Loci
RILs: Recombinant inbred lines
BRM: Block Regression Mapping
ΔSNP: Delta-SNP index
t-ΔSNP: Tricube delta-SNP index
EDm: Euclidean Distance at marker level
ED_100_^4^: Euclidean Distance smoothed for groups of 100 consecutive markers
AFDexp: Allele Frequency Difference smoothed according to Huang et al*.* 2019
LOD: LOD score at marker level according to Zhang et al*.* 2019
SmLOD: Smoothed LOD according to Zhang et al*.* 2019
