# Supplementary material for: Evaluation of nine statistics to identify QTLs in bulk segregant analysis using next generation sequencing approaches
Source: BMC Genomics. 2022 Jul 6;23:490. doi: 10.1186/s12864-022-08718-y (PMC9258084; doi:10.1186/s12864-022-08718-y)
Supplement: Supplementary file 1 — Additional file 1. Supplementary figures showing the results of simulations of a NGS-based BSA case study using different statistics for the detection of a single QTL in a model chromosome at three recombination rates (λ=0.90, λ=1.30 and λ=2.15). Figure S1. Result of simulations using binomial distribution in the simulation of sequencing noise and a QTL effect equivalent to 20% of the phenotypic variance (k=1). Figure S2. Result of simulations using real data from rice to add sequencing noise and a QTL effect equivalent to 20% of the phenotypic variance (k=1). Figure S3. Result of simulations using binomial distribution in the simulation of sequencing noise and a QTL effect equivalent to 5.9% of the phenotypic variance (k=0.5). Figure S4. Result of simulations using real data from rice to add sequencing noise and a QTL effect equivalent to 5.9% of the phenotypic variance (k=0.5) [file 12864_2022_8718_MOESM1_ESM.pdf]

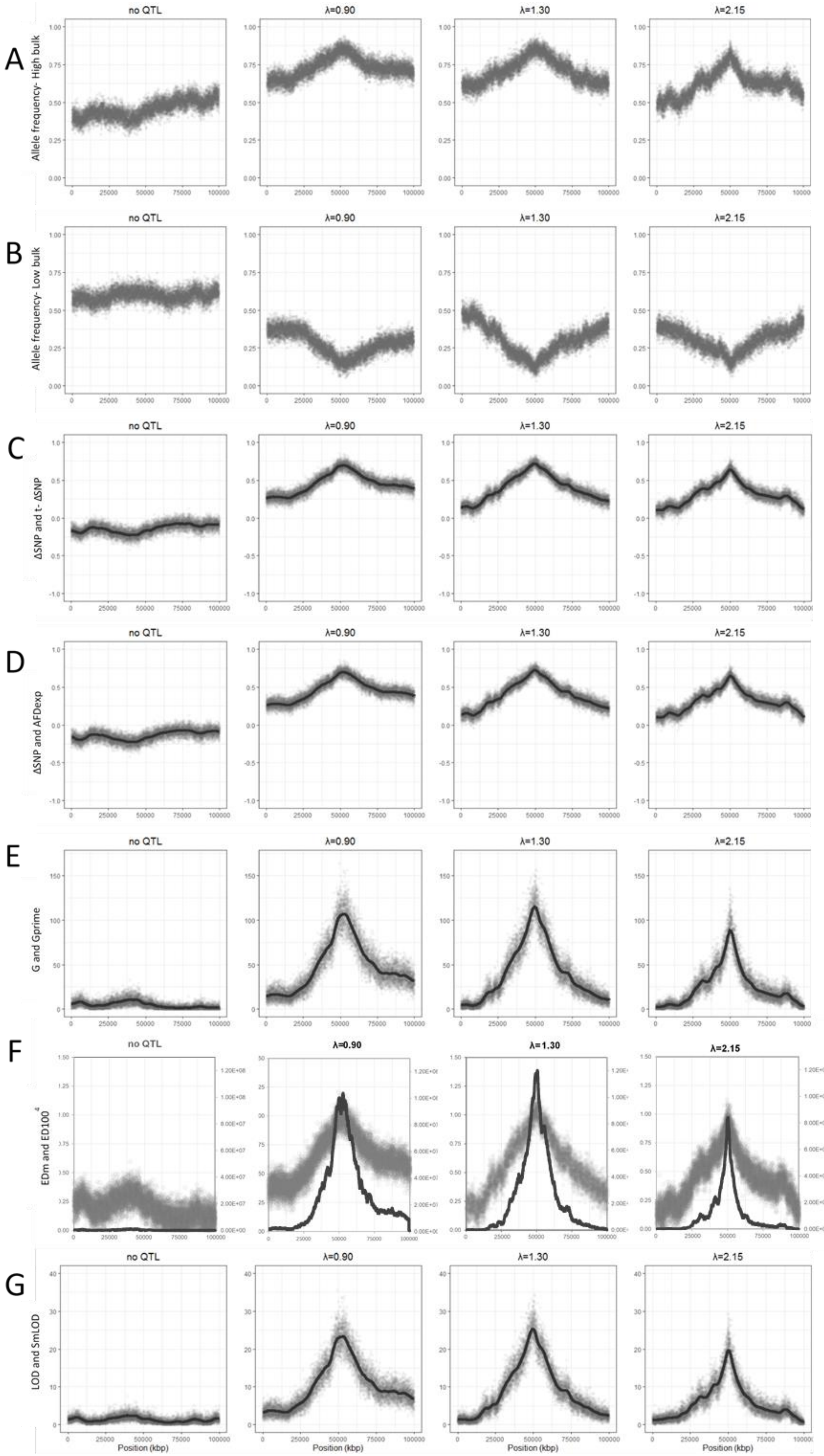
A
B
C
D
E
F
G
no QTL
λ=0.90
λ=1.30
λ=2.15
Allele frequency- High bulk
Allele frequency- Low bulk
ΔSNP and t- ΔSNP
ΔSNP and AFDexp
G and Gprime
EDm and ED100^4
LOD and SmLOD
Position (kbp)

Figure S1: Simulation of a NGS-based BSA case study using different statistics for the detection of a single QTL in a model chromosome at three recombination rates (λ=0.90, λ=1.30 and λ=2.15). Results of simulations using binomial distribution in the simulation of sequencing noise and QTL effect equivalent to 20% of the phenotypic variance (k=1). The frequency of the alternate allele for each marker position is represented in two contrasted pools of segregant lines displaying high phenotype (A) and low phenotype (B). In each graph, grey dots correspond to allele frequency or statistics value at marker level; the black line shows the smoothed value of the statistic. The difference in allele frequency (ΔSNP) between pools and the smoothed statistics for window corresponding to 3Mbp (line) lead to the detection of a QTL simulated in the middle of the 100 Mbp model chromosome using t- ΔSNP (C) and AFDexp (D). QTL detection is shown based on G and Gprime statistics (E), EDm and $ED100^4$ statistics (F) and log likelihood LOD and Smooth-LOD (G) for different species with different value of their recombination ratio (λ): pearl millet (λ=0.90), rice (λ=1.30) and foxtail millet (λ=2.15). The first graph for each row show the results in absence of QTL effect.

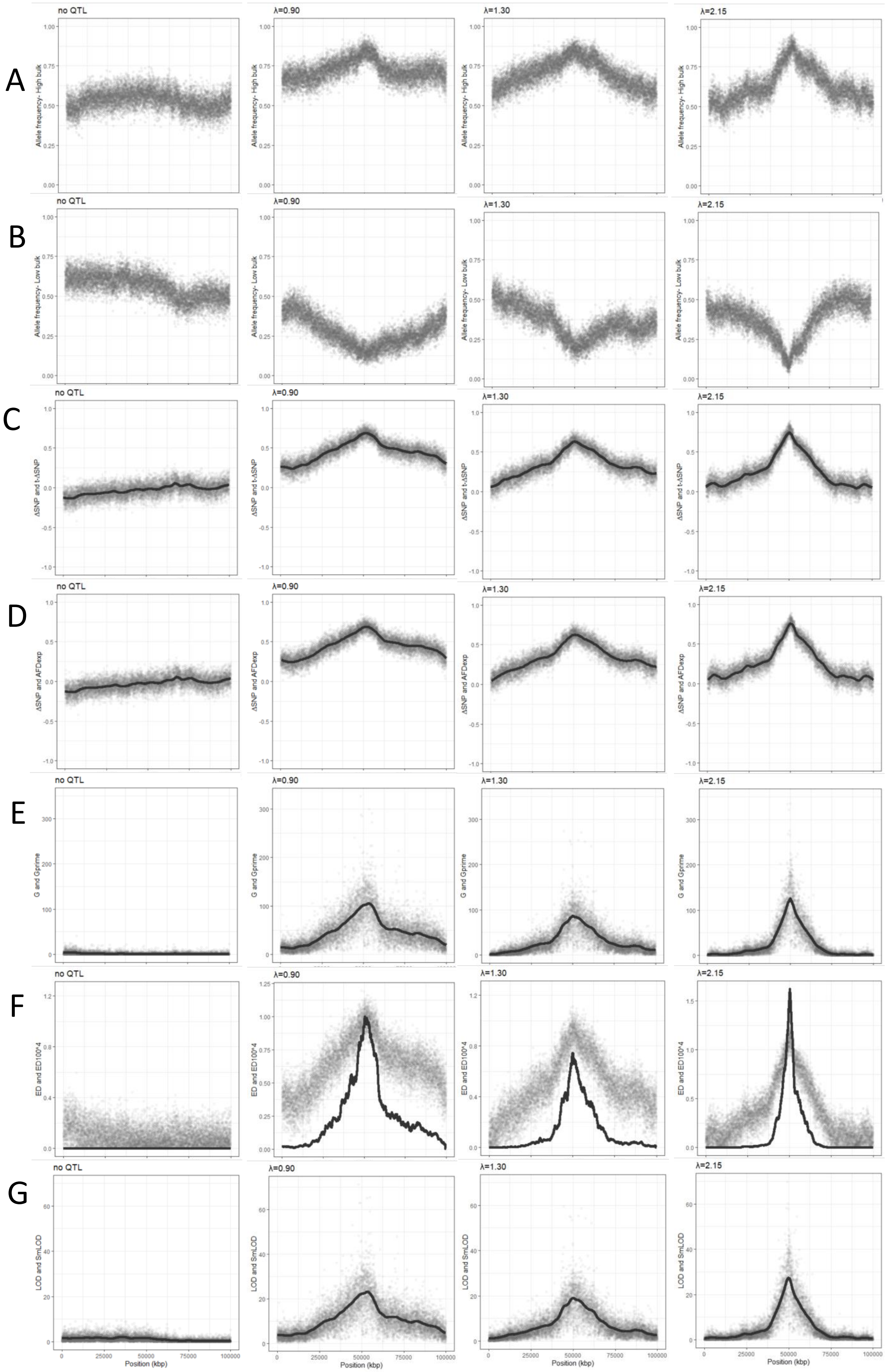

A
B
C
D
E
F
G
no QTL
λ=0.90
λ=1.30
λ=2.15
Allele frequency- High bulk
Allele frequency- Low bulk
ΔSNP and t-ΔSNP
ΔSNP and AFDexp
G and Gprime
ED and ED100^4
LOD and SmLOD
Position (kbp)

Figure S2: Simulation of a NGS-based BSA case study using different statistics for the detection of a single QTL in a model chromosome at three recombination rates (λ=0.90, λ=1.30 and λ=2.15). Results of simulations real data from rice to add sequencing noise and QTL effect equivalent to 20% of the phenotypic variance (k=1). The frequency of the alternate allele for each marker position is represented in two contrasted pools of segregant lines displaying high phenotype (A) and low phenotype (B). In each graph, grey dots correspond to allele frequency or statistics value at marker level; the black line shows the smoothed value of the statistic. The difference in allele frequency (ΔSNP) between pools and the smoothed statistics for window corresponding to 3Mbp (line) lead to the detection of a QTL simulated in the middle of the 100 Mbp model chromosome using t- ΔSNP (C) and AFDexp (D). QTL detection is shown based on G and Gprime statistics (E), EDm and $ED100^4$ statistics (F) and log likelihood LOD and Smooth-LOD (G) for different species with different value of their recombination ratio (λ): pearl millet (λ=0.90), rice (λ=1.30) and foxtail millet (λ=2.15). The first graph for each row shows the results in absence of QTL effect.

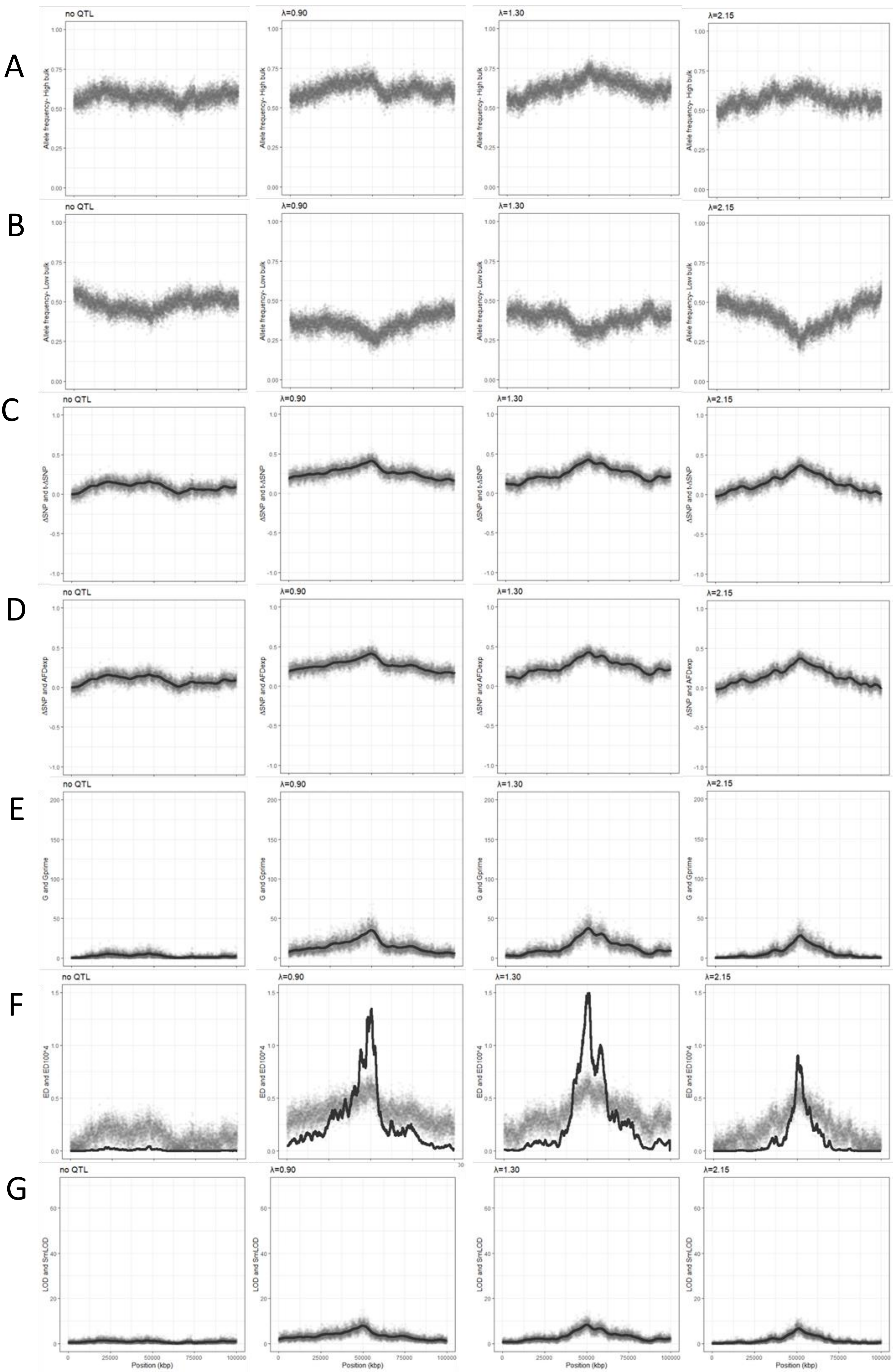
A
B
C
D
E
F
G
no QTL
λ=0.90
λ=1.30
λ=2.15
Allele frequency- High bulk
Allele frequency- Low bulk
ΔSNP and t-ΔSNP
ΔSNP and AFDexp
G and Gprime
ED and ED100^4
LOD and SmLOD
Position (kbp)
0
25000
50000
75000
100000

Figure S3: Simulation of a NGS-based BSA case study using different statistics for the detection of a single QTL in a model chromosome at three recombination rates (λ=0.90, λ=1.30 and λ=2.15). Results of simulations using binomial distribution to simulate sequencing noise and QTL effect equivalent to 5.9% of the phenotypic variance (k=0.5). The frequency of the alternate allele for each marker position is represented in two contrasted pools of segregant lines displaying high phenotype (A) and low phenotype (B). In each graph, grey dots correspond to allele frequency or statistics value at marker level; the black line shows the smoothed value of the statistic. The difference in allele frequency (ΔSNP) between pools and the smoothed statistics for window corresponding to 3Mbp (line) lead to the detection of a QTL simulated in the middle of the 100 Mbp model chromosome using t- ΔSNP (C) and AFDexp (D). QTL detection is shown based on G and Gprime statistics (E), EDm and ED100[4] statistics (F) and log likelihood LOD and Smooth-LOD (G) for different species with different value of their recombination ratio (λ): pearl millet (λ=0.90), rice (λ=1.30) and foxtail millet (λ=2.15). The first graph for each row shows the results in absence of QTL effect.

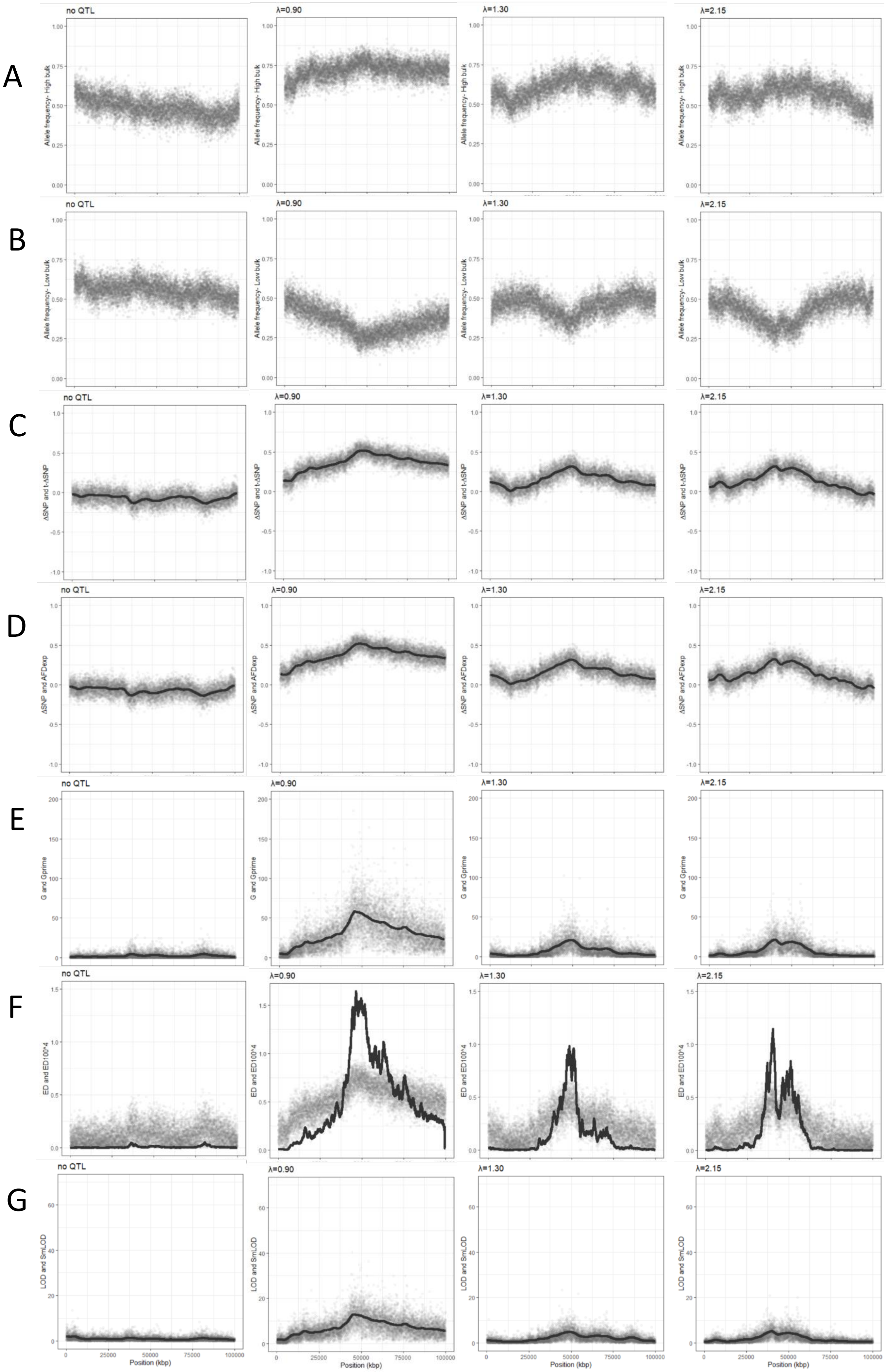

A
B
C
D
E
F
G
no QTL
λ=0.90
λ=1.30
λ=2.15
Allele frequency- High bulk
Allele frequency- Low bulk
ΔSNP and t-ΔSNP
ΔSNP and AFDexp
G and Gprime
ED and ED100^4
LOD and SmLOD
Position (kbp)

Figure S4: Simulation of a NGS-based BSA case study using different statistics for the detection of a single QTL in a model chromosome at three recombination rates (λ=0.90, λ=1.30 and λ=2.15). Results of simulations using real data from rice to add sequencing noise and QTL effect equivalent to 5.9% of the phenotypic variance (k=0.5). The frequency of the alternate allele for each marker position is represented in two contrasted pools of segregant lines displaying high phenotype (A) and low phenotype (B). In each graph, grey dots correspond to allele frequency or statistics value at marker level; the black line shows the smoothed value of the statistic. The difference in allele frequency (ΔSNP) between pools and the smoothed statistics for window corresponding to 3Mbp (line) lead to the detection of a QTL simulated in the middle of the 100 Mbp model chromosome using t- ΔSNP (C) and AFDexp (D). QTL detection is shown based on G and Gprime statistics (E), EDm and $ED100^4$ statistics (F) and log likelihood LOD and Smooth-LOD (G) for different species with different value of their recombination ratio (λ): pearl millet (λ=0.90), rice (λ=1.30) and foxtail millet (λ=2.15). The first graph for each row shows the results in absence of QTL effect.
