# Supplementary material for: Evaluation of nine statistics to identify QTLs in bulk segregant analysis using next generation sequencing approaches
Source: BMC Genomics. 2022 Jul 6;23:490. doi: 10.1186/s12864-022-08718-y (PMC9258084; doi:10.1186/s12864-022-08718-y)
Supplement: Supplementary file 2 — Additional file 2. Supplementary figure and tables showing the results of comparative analysis of BSA statistical methods and their accuracy in locating QTLs in 1,000 simulations. Figure S1. Comparative analysis of the inferred position of simulated QTLs using rice real data to simulate sequencing noise. Table S1. Pairwise comparison of different statistical approaches inferring the position of a simulated QTL. The positions used correspond to 1,000 simulations including binomial distribution to add sequencing noise (Figure 3). Table S2. Summary statistics of the absolute genetic distance (kb) between the simulated QTL and the QTL position retrieved for 1,000 simulations run with each statistic in each model chromosome (Figure 3). Table S3. Pairwise comparison of different statistical approaches inferring the position of a simulated QTL. The positions used correspond to 1,000 simulations using rice real data to add sequencing noise (Additional file 2- Figure S1). Table S4. Summary statistics of the absolute genetic distance (kb) between the simulated QTL and the QTL position retrieved for 1,000 simulations with each statistic in each model chromosome (Additional file 2- Figure S1). [file 12864_2022_8718_MOESM2_ESM.pdf]

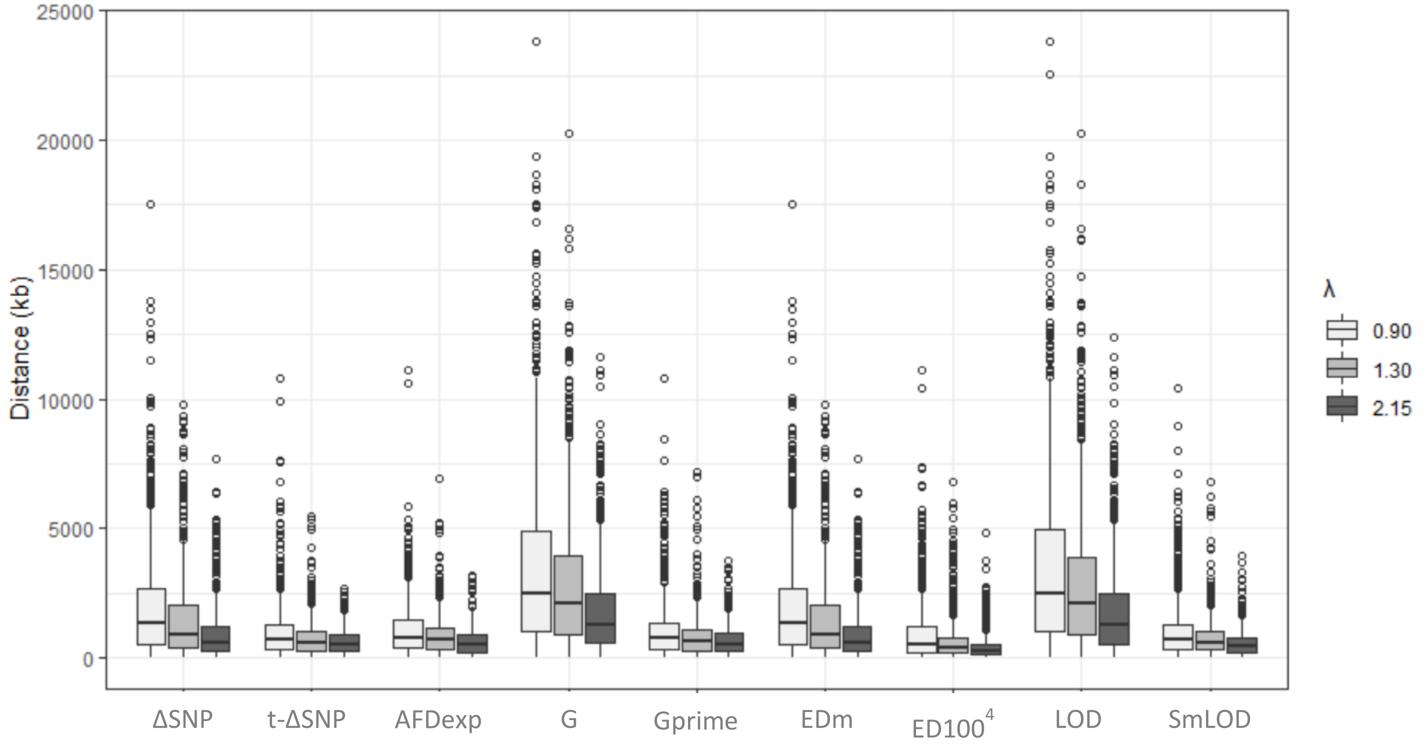

Figure S1. Comparative analysis of the inferred position of simulated QTLs using rice real data in the simulation of sequencing noise. To assess the accuracy of QTL detection using nine statistics based on calculation of ΔSNP, G, ED and LOD at the marker level in three case studies of recombination rate (λ), we plot the distance to the simulated QTL in kb. Our calculations were based on differences in allele frequency (ΔSNP), G-statistics (G), Euclidian distance (ED) and log likelihood (LOD). Methods use either data at the SNP level (ΔSNP, G, EDm, LOD) or a smooth value across several SNPs (t-ΔSNP, AFDexp, Gprime, $ED100^4$, SmLOD). The boxplots represent the range of detection of QTLs in one thousand simulations using each method. Distance corresponds to the absolute genetic distance between the simulated QTL and the QTL position retrieved with each method.

Table S1. Pairwise comparison of different statistical approaches inferring the position of a simulated QTL. The tables show the p-value significance level of the Wilcoxon rank sum test calculated for the log value of the genetic distance between the initial QTL position and the QTL peak position obtained with different statistical approaches. Pairwise comparisons of the methods are shown for each model chromosome. *** *p*<0.001, ** *p*<0.01, * *p*<0.05, ns: not significant.

Pearl millet (λ=0.90)

	ΔSNP	t-ΔSNP	AFDexp	G	Gprime	EDm	ED100[4]	LOD
t-ΔSNP	***	-	-	-	-	-	-	-
AFDexp	***	ns	-	-	-	-	-	-
G	ns	***	***	-	-	-	-	-
Gprime	***	ns	ns	***	-	-	-	-
EDm	ns	***	***	ns	***	-	-	-
ED100[4]	***	***	***	***	***	***	-	-
LOD	*	***	***	ns	***	*	***	-
SmLOD	***	**	***	***	*	***	***	***

Rice (λ=1.30)

	ΔSNP	t-ΔSNP	AFDexp	G	Gprime	EDm	ED100[4]	LOD
t-ΔSNP	***	-	-	-	-	-	-	-
AFDexp	***	ns	-	-	-	-	-	-
G	ns	***	***	-	-	-	-	-
Gprime	***	ns	ns	***	-	-	-	-
EDm	ns	***	***	ns	***	-	-	-
ED100[4]	***	***	***	***	***	***	-	-
LOD	ns	***	***	ns	***	ns	***	-
SmLOD	***	***	**	***	**	***	***	***

Foxtail millet (λ=2.15)

	ΔSNP	t-ΔSNP	AFDexp	G	Gprime	EDm	ED100[4]	LOD
t-ΔSNP	ns	-	-	-	-	-	-	-
AFDexp	ns	ns	-	-	-	-	-	-
G	ns	ns	ns	-	-	-	-	-
Gprime	ns	ns	ns	ns	-	-	-	-
EDm	ns	ns	ns	ns	ns	-	-	-
ED100[4]	***	***	***	***	***	***	-	-
LOD	ns	ns	ns	ns	ns	ns	***	-
SmLOD	***	***	***	*	***	***	***	ns

Table S2. Summary statistics of the absolute genetic distance (kb) between the simulated QTL and the QTL position retrieved for one thousand simulations run with each statistic in each model chromosome. Pearl millet (λ=0.90), rice (λ=1.30) and foxtail millet (λ=2.15).

ΔSNP	model	Min.	1st Qu.	Median	Mean	3rd Qu.	Max.
	λ=0.90	0	450	1095	1729	2340	16540
	λ=1.30	0	347.5	885	1233.2	1720	9570
	λ=2.15	0	200	510	777.5	1020	6190

t-ΔSNP	model	Min.	1st Qu.	Median	Mean	3rd Qu.	Max.
	λ=0.90	0	340	745	965.1	1290	9670
	λ=1.30	0	330	650	798.8	1090	6110
	λ=2.15	0	230	530	628.7	880	4490

AFDexp	model	Min.	1st Qu.	Median	Mean	3rd Qu.	Max.
	λ=0.90	0	350	770	1158	1370	90750
	λ=1.30	0	300	650	947.9	1080	79620
	λ=2.15	0	230	520	822	932.5	61640

G	model	Min.	1st Qu.	Median	Mean	3rd Qu.	Max.
	λ=0.90	0	420	1000	1594	2130	19420
	λ=1.30	0	310	790	1143	1540	8990
	λ=2.15	0	190	460	675.4	920	6080

Gprime	model	Min.	1st Qu.	Median	Mean	3rd Qu.	Max.
	λ=0.90	0	340	740	955.1	1272.5	9670
	λ=1.30	0	317.5	650	796.7	1090	6110
	λ=2.15	0	230	530	629.6	880	4490

EDm	model	Min.	1st Qu.	Median	Mean	3rd Qu.	Max.
	λ=0.90	0	450	1095	1729	2340	16540
	λ=1.30	0	347.5	885	1233.2	1720	9570
	λ=2.15	0	200	510	777.5	1020	6190

ED100^4	model	Min.	1st Qu.	Median	Mean	3rd Qu.	Max.
	λ=0.90	0	210	480	863.7	1050	10760
	λ=1.30	0	170	360	608.5	770	6390
	λ=2.15	0	120	260	402.7	510	4050

LOD	model	Min.	1st Qu.	Median	Mean	3rd Qu.	Max.
	λ=0.90	0	400	970	1598	2130	17780
	λ=1.30	0	310	815	1141	1540	8990
	λ=2.15	0	180	450	675.6	910	6080

SmLOD	model	Min.	1st Qu.	Median	Mean	3rd Qu.	Max.
	λ=0.90	0	310	670	894.1	1140	16340
	λ=1.30	0	270	560	705.9	950	5740
	λ=2.15	0	210	420	533.3	760	4740

Table S3. Pairwise comparison of different statistical approaches inferring the position of a simulated QTL in simulations using real data from rice to add sequencing noise. The tables show the p-value significance level of the Wilcoxon rank sum test calculated for the log value of the genetic distance between the initial QTL position and the QTL peak position obtained with different statistical approaches. Pairwise comparisons of the methods are shown for each model chromosome. *** p<0.001, ** p<0.01, * p<0.05, ns: not significant.

Pearl millet (λ= 0.90)

	ΔSNP	t-ΔSNP	AFDexp	G	Gprime	EDm	ED100^4	LOD
t-ΔSNP	***	-	-	-	-	-	-	-
AFDexp	***	*	-	-	-	-	-	-
G	***	***	***	-	-	-	-	-
Gprime	***	ns	ns	***	-	-	-	-
EDm	ns	***	***	***	***	-	-	-
ED100^4	***	***	***	***	***	***	-	-
LOD	***	***	***	ns	***	***	***	-
SmLOD	***	ns	ns	***	ns	***	***	***

Rice (λ= 1.30)

	ΔSNP	t-ΔSNP	AFDexp	G	Gprime	EDm	ED100^4	LOD
t-ΔSNP	***	-	-	-	-	-	-	-
AFDexp	***	***	-	-	-	-	-	-
G	***	***	***	-	-	-	-	-
Gprime	***	ns	*	***	-	-	-	-
EDm	ns	***	***	***	***	-	-	-
ED100^4	***	***	***	***	***	***	-	-
LOD	***	***	***	ns	***	***	***	-
SmLOD	***	ns	***	***	ns	***	***	***

Foxtail millet (λ= 2.15)

	ΔSNP	t-ΔSNP	AFDexp	G	Gprime	EDm	ED100^4	LOD
t-ΔSNP	***	-	-	-	-	-	-	-
AFDexp	***	ns	-	-	-	-	-	-
G	***	***	***	-	-	-	-	-
Gprime	**	ns	ns	***	-	-	-	-
EDm	ns	***	***	***	**	-	-	-
ED100^4	***	***	***	***	***	***	-	-
LOD	***	***	***	ns	***	***	***	-
SmLOD	***	**	*	***	***	***	***	***

Table S4. Summary statistics of the absolute genetic distance (kb) between the simulated QTL and the QTL position retrieved for one thousand simulations with each statistic in each model chromosome. Simulations consider rice real data in the simulation of sequencing noise. Pearl millet (λ=0.90), rice (λ=1.30) and foxtail millet (λ=2.15).

ΔSNP	model	Min.	1st Qu.	Median	Mean	3rd Qu.	Max.
	λ=0.90	0	520	1355	1993.28	2670	17500
	λ=1.30	0	380	900	1470.9	2050	9790
	λ=2.15	0	240	580	901.25	1210	7670

t-ΔSNP	model	Min.	1st Qu.	Median	Mean	3rd Qu.	Max.
	λ=0.90	0	330	710	964.28	1265	10810
	λ=1.30	0	260	580	723.25	1000	5450
	λ=2.15	0	268	510	617.77	892	2700

AFDexp	model	Min.	1st Qu.	Median	Mean	3rd Qu.	Max.
	λ=0.90	0	380	800	1047.15	1470	11090
	λ=1.30	0	340	700	850.58	1150	6910
	λ=2.15	0	230	510	636.82	930	3170

G	model	Min.	1st Qu.	Median	Mean	3rd Qu.	Max.
	λ=0.90	0	1040	2485	3477.86	4932	23830
	λ=1.30	0	890	2120	2842.31	3920	20230
	λ=2.15	0	570	1280	1805.31	2460	11610

Gprime	model	Min.	1st Qu.	Median	Mean	3rd Qu.	Max.
	λ=0.90	0	360	770	1045.68	1380	10810
	λ=1.30	0	280	620	810.95	1115	7200
	λ=2.15	0	270	530	653.2	940	3750

EDm	model	Min.	1st Qu.	Median	Mean	3rd Qu.	Max.
	λ=0.90	0	520	1355	1993.16	2670	17500
	λ=1.30	0	380	900	1470.9	2050	9790
	λ=2.15	0	240	580	901.25	1210	7670

$ED100^4$	model	Min.	1st Qu.	Median	Mean	3rd Qu.	Max.
	λ=0.90	0	230	510	912.9	1200	11120
	λ=1.30	0	190	390	635.82	770	6780
	λ=2.15	0	130	280	428.45	520	4840

LOD	model	Min.	1st Qu.	Median	Mean	3rd Qu.	Max.
	λ=0.90	0	1070	2510	3510.59	4980	23830
	λ=1.30	0	892	2080	2854.48	3902	20230
	λ=2.15	0	552	1280	1818.98	2460	12360

SmLOD	model	Min.	1st Qu.	Median	Mean	3rd Qu.	Max.
	λ=0.90	0	360	730	1024.19	1270	10420
	λ=1.30	0	302	560	757.72	1010	6830
	λ=2.15	0	220	455	572.25	810	3950
