# Supplementary material for: Evaluation of nine statistics to identify QTLs in bulk segregant analysis using next generation sequencing approaches
Source: BMC Genomics. 2022 Jul 6;23:490. doi: 10.1186/s12864-022-08718-y (PMC9258084; doi:10.1186/s12864-022-08718-y)
Supplement: Supplementary file 3 — Additional file 3. Table showing the confidence intervals of each of the statistics. The calculation is based on 10,000 simulations for each model chromosome. [file 12864_2022_8718_MOESM3_ESM.pdf]

Additional file 3: Confidence intervals of each of the statistics calculated using ten thousand simulations

	Model chromosome		
	λ=0.90	λ=1.30	λ=2.15
deltaSNP			
97.50%	0.1674	0.1717	0.1708
2.50%	-0.1709	-0.1682	-0.1671
tricubeDelta			
97.50%	0.1377	0.1360	0.1349
2.50%	-0.1400	-0.1340	-0.1307
AFDexp			
97.50%	0.1366	0.1358	0.1339
2.50%	-0.1381	-0.1362	-0.1359
G			
95%	5.8886	5.7361	5.8407
Gprime			
95%	4.2433	4.2145	4.2668
EDm			
95%	0.2408	0.2404	0.2420
$ED100^4$			
95%	153522.5	137470.6	158207.1
LOD			
95%	1.9775	1.9734	1.9893
SmLOD			
95%	1.4866	1.4974	1.4585
