# Supplementary material for: Evaluation of nine statistics to identify QTLs in bulk segregant analysis using next generation sequencing approaches
Source: BMC Genomics. 2022 Jul 6;23:490. doi: 10.1186/s12864-022-08718-y (PMC9258084; doi:10.1186/s12864-022-08718-y)
Supplement: Supplementary file 4 — Additional file 4. General summary of the main steps of the simulations. [file 12864_2022_8718_MOESM4_ESM.pdf]

Additional file 4. Main steps of the simulations

1. Define general settings for simulations and the matrix where output result will be saved after each simulation loop when nbsim>1

Settings	
test	Number of statistical tests
nbsim	Number of simulations
nbchr	Chromosome number (=population size x 2)
loci	Number of loci per chromosome
qtleffect	Effect of the QTL

Matrices	
QTLpos	Matrix compiling QTL initial position and the one retrieved with each statistical test in total number of simulations (nbsim)
CIs	Matrix grouping the statistical values in absence of QTL effect in total number of simulations (nbsim). The matrix will be subsequently used in the calculation of confidence intervals.

Start of nbsim simulations {

2. Simulate the vector of recombination events (q) following a Poisson distribution with a frequency of recombination of $\lambda = 0.90$ for pearl millet, $\lambda = 1.30$ for rice and $\lambda = 2.15$ for foxtail millet.

3. Define the genotype matrix (geno). 0s and 1s will code the reference and alternate parental alleles respectively.

4. Define a normally distributed phenotype - N(0,1) - for nbchr/2 individuals.

5. Simulate the QTL position and calculate the QTL additive effect. QTL position fixed for single case study (middle locus of model chromosome) or random allocated position if nbsim>1.

6. Establish the bulks of contrasted lines (H and L) and group the genotype information for each bulk

7. Calculate the depth of sequencing and allele frequencies (P) at each marker position.
   Arrange the data in format for QTLseqr.

8. Add sequencing noise according to binomial function B(n, P)

9. Run the five statistical tests for BSA and retrieve output results when nbsim >1

Statistical test		R package / GitHub script	Comments
At SNP level	Smoothed for Window (W)		
ΔSNP	t-ΔSNP	QTLseqr	Allele counts of each bulk need to follow the QTLseqr name format
G	Gprime		
EDm	ED_{100}^4		
LOD	SmLOD	QTG script for SmoothLOD in GitHub	Load the LOD function before simulations loop
ΔSNP	AFDexp	BRM script from GitHub	Allele counts of each bulk need to follow the BRM script name format<br>$u_{\alpha/2}$ was defined for each model chromosome: 3.43 for pearl millet; 3.65 for rice and 3.71 for foxtail millet.

} End of nbsim simulations

10. Save the matrix with output results in csv and transfer to computer
